# Cannabidiol modulation of oxidative stress and signalling

**DOI:** 10.1042/NS20200080

**Published:** 2021-08-24

**Authors:** Sónia R. Pereira, Becky Hackett, David N. O’Driscoll, Melody Cui Sun, Eric J. Downer

**Affiliations:** Discipline of Physiology, School of Medicine, Trinity Biomedical Sciences Institute, Trinity College Dublin, Dublin 2, Ireland

**Keywords:** Cannabidiol, Cannabinoids, Oxidative signalling, Oxidative stress, Reactive oxygen species

## Abstract

Cannabidiol (CBD), one of the primary non-euphoric components in the *Cannabis sativa* L. plant, has undergone clinical development over the last number of years as a therapeutic for patients with Lennox-Gastaut syndrome and Dravet syndromes. This phytocannabinoid demonstrates functional and pharmacological diversity, and research data indicate that CBD is a comparable antioxidant to common antioxidants. This review gathers the latest knowledge regarding the impact of CBD on oxidative signalling, with focus on the proclivity of CBD to regulate antioxidants and control the production of reactive oxygen species. CBD is considered an attractive therapeutic agent for neuroimmune disorders, and a body of literature indicates that CBD can regulate redox function at multiple levels, with a range of downstream effects on cells and tissues. However, pro-oxidant capacity of CBD has also been reported, and hence caution must be applied when considering CBD from a therapeutic standpoint. Such pro- and antioxidant functions of CBD may be cell- and model-dependent and may also be influenced by CBD dose, the duration of CBD treatment and the underlying pathology.

## Introduction

Production of reactive oxygen species (ROS) is commonly associated with oxidative stress and its pathological role in inflammatory diseases such as multiple sclerosis (MS), rheumatoid arthritis (RA), atherosclerosis and inflammatory bowel disease (IBD) [1–5]. During infection, immune cells produce ROS via the NADPH oxidase 2 (NOX2) complex as a mechanism to eradicate pathogens [6]. When NOX2-generated ROS production is dysregulated due to mutations in NOX2 complex proteins, this can result in defective phagocyte function characterized by severe and recurrent infections defined as chronic granulomatous disease (CGD) [7]. Moreover, ROS produced in the phagosome to activate proteolytic enzymes can escape the immune cell, thus damaging the surrounding tissue [8]. Despite this, it is clear that a deficiency in ROS production has the proclivity to aggravate disease processes [9]. In addition, leukocytes isolated from individuals with chronic MS produce less superoxide than those with a milder disease [10], and a similar scenario has been identified in Guillain–Barré syndrome (a subtype of acute inflammatory demyelinating polyneuropathy), where evidence suggests that leukocytes produce lower levels of oxygen radicals in the most severe cases of disease [11]. Taken together, these findings support a complex role of ROS in regulating inflammation in disease.

In recent years, cannabinoid molecules, such as cannabidiol (CBD) and Δ^9^-tetrahydrocannabinol (Δ^9^-THC), have drawn attention due to their anti-inflammatory, antioxidant and neuroprotective properties [12,13]. The most well-described targets for cannabinoids are their specific receptors, the cannabinoid receptors CB_1_ and CB_2_ [14,15], but their pharmacological actions are not solely limited to these receptors. Indeed, cannabinoids are lipophilic and certain cannabinoids have also been shown to target a wide range of receptors, including the peroxisome proliferator-activated receptors (PPARs), the transient receptor potential cation channel subfamily V member 1 (TRPV1), G-protein-coupled receptor 55 (GPR55), the 5-hydroxytryptamine receptor subtype 1A (5-HT1A), glycine α1 and α1β receptors, in addition to ion channels (Ca^2+^) and enzymes such as the adenosine membrane transporter phospholipase A2, lipoxygenase (LO) and cyclooxygenase-2 (COX-2) [16–22]. Depending both on the cannabinoid structure and cell/tissue targeted, the pharmacological effects of cannabinoids may vary.

Overall, cannabinoids have been shown to possess therapeutic efficacy in several inflammatory and neuronal diseases [23]. Given that the production of ROS is an intrinsic feature of neuroinflammation and peripheral immune responses, this review aims to gather the latest knowledge on the action of cannabinoids on oxidative signalling, with focus on the phytocannabinoid CBD. CBD is selected for review given recent advances in its therapeutic development [24–26].

## Oxidative signalling and stress

The production and maintenance of controlled levels of intracellular ROS has a key role in several physiological functions, including the maintainance of redox homeostasis, cell cycle signalling and hormone production [27,28]. When present, ROS can regulate several signalling pathways by reacting with transcription factors and genes, modifying their structure and thus their function. Hence, ROS can modulate gene expression patterns and signalling proteins related to the stress response and cell survival mechanisms [29]. It is when this homeostasis is impaired, by either an overproduction of ROS or inefficient ROS scavenging mechanisms, that oxidative stress ensues, promoting cellular damage, lipid peroxidation, DNA modifications and enzyme inactivation, and when persistent, can ultimately lead to cell death and tissue destruction [30–32]. This rationale has been the basis for the development of several anticancer drugs [33–35].

## ROS

**Figure 1:**
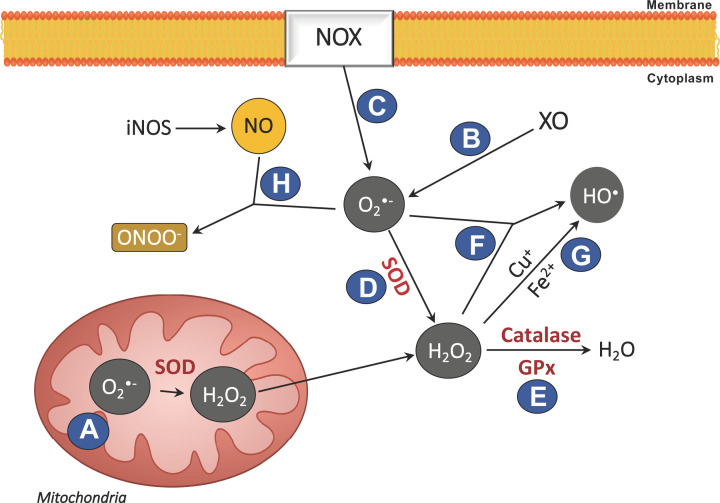
Modulation of cellular ROS The majority of ROS originate from various sources within the cell. Production is via (**A**) the mitochondrial electron transport chain, (**B**) the enzymatic reaction catalysed by XO and (**C**) by NOX. Endogenous antioxidant mechanisms are exerted via (**D**) SOD conversion of O_2_^•−^ to H_2_O_2_, followed by conversion to water by (**E**) CAT and/or GPx. When ROS production exceeds the endogenous antioxidant mechanisms capacity, (**F** and **G**) H_2_O_2_ may react with O_2_^•−^ to form HO^•^ (Haber–Weiss and Fenton reactions). (**H**) Oxygen radicals can also react with NO to generate ONOO-.

ROS represent a group of unstable oxygen radicals and molecules with strong oxidizing properties. Once formed, ROS are converted to other oxidative species or eliminated by the antioxidant mechanisms of the cell [29,36]. The most common ROS are the superoxide anion (O_2_^•−^), hydrogen peroxide (H_2_O_2_) and the hydroxyl radical (HO^•^) [29]. There are three major cellular mechanisms of ROS production as described in Figure 1: (A) ROS are produced via the mitochondrial electron transport chain, in complex I and II; (B) production via the enzymatic reaction catalysed by the enzyme xanthine oxidase (XO); and (C) by NOX as a defence against pathogens [37–39]. To maintain the levels of ROS under control, the enzyme superoxide dismutase (SOD) converts O_2_^•−^ into H_2_O_2_ (D), which is then converted into water, a reaction catalysed by catalase (CAT) and/or glutathione peroxidase (GPx) (E) [40,41]. However, during periods of high ROS generation, H_2_O_2_ may cross cell membranes and react with O_2_^•−^ and metal cations (Fe^2+^ or Cu^+^) to form HO^•^ (Fenton and Haber–Weiss reactions) (F, G) [42]. Furthermore, oxygen radicals can also react with nitrogen species, namely nitric oxide (NO) (H). The reaction of O_2_^•−^ with NO can generate the production of peroxynitrite (ONOO^−^) (H) [43], a particularly reactive radical that may promote generalized oxidative/nitrosative damage, including DNA fragmentation and lipid degradation.

## NOX2 production of ROS

One of the main cellular sources of ROS is the NOX2 complex, which belongs to a family of NADPH oxidases. NOX are transmembrane enzymes involved in the electron transport across biological membranes by oxidizing intracellular NADPH = NADH, while reducing molecular oxygen into O_2_^•−^ anions [37]. Among the seven NOX members identified to date, NOX2 has the ability to produce an oxidative burst to eliminate pathogens [44]. The NOX2 complex consists in a transmembrane catalytic core, a heterodimer containing gp91phox and the protein p22phox. On the cytosolic side of the membrane, the proteins p47phox, p67phox, p40phox and Rac (a small GTP-binding protein) regulate the complex activity [37,45].

Antigen-presenting cells can produce ROS upon activation via the NOX2 complex [6,46]. Interestingly, gp91phox is also expressed in bone marrow and thymus, contributing to the role of NOX2-derived ROS in B-/T-cell development and maturation [47,48]. Although phagocytes express high levels of the NOX2 complex, components of this complex are also found in nonphagocytic cells, such as cardiomyocytes, endothelial cells and muscle cells [49–51]. In resting cells, the NOX2 complex remains inactive, and upon exposure to certain stimuli, the complex becomes primed or activated [52]. NOX2 priming prepares the complex to produce ROS, initiating a weak oxidative response without eliciting an oxidative burst. This can occur upon stimulation by pro-inflammatory cytokines (e.g. tumour necrosis factor-α [TNF-α] and interleukin [IL]-1β, toll-like receptor [TLR] agonists, e.g. lipopolysaccharide [LPS], flagellin), OONO^−^ and proteases. The priming induces a partial phosphorylation of p47phox that alters its conformation, thus decreasing its autoinhibition and facilitating its translocation and interaction with the NOX2 enzymatic core. This mechanism exerts tight control over NOX2 activation to prevent unintended O_2_^•−^ release [53,54]. Full activation of the NOX2 complex requires additional stimuli, such as phorbol 12-myristate 13-acetate, a protein kinase C activator and formyl-methionyl-leucyl phenylalanine, which acts via the formylpeptide receptor. Upon activation, NOX2 increases the production of superoxide [55], and therefore, mutations in any of the components of NOX2 dysregulate its activity and thus ROS signalling [7,56].

## Antioxidant mechanisms

Cellular antioxidant mechanisms regulate ROS signalling and furthermore prevent/reduce the oxidation of unintended molecules. Antioxidant mechanisms include enzymes such as CAT, SOD, GPx, thioredoxin and peroxiredoxin, in addition to the radical scavengers such as reduced glutathione (GSH) [40,41,57,58]. As mentioned previously, CAT catalyses the conversion of H_2_O_2_ to water (Figure 1), and although this enzyme is highly expressed during inflammation, the GPx enzyme has even higher affinity to the H_2_O_2_ radical. GPx activity depends on the GSH ability to be oxidized to glutathione disulfide (GSSG) in the presence of NADPH, thus functioning as a proton donor. The GSH pool is afterwards replenished by both its regeneration from GSSG mediated by GSH reductase and *de novo* synthesis [59,60]. Additionally, dietary antioxidant molecules are also useful at maintaining the level of ROS by directly scavenging ROS, namely tocopherols (vitamin E) and ascorbic acid (vitamin C) [61,62].

More research is still needed to achieve a deeper understanding on the specific roles for each particular oxygen radical. Current methodologies are limited in their ability to measure isolated radicals but rather a group of radicals, making it difficult to differentiate amongst them. What is known today in the field is based on the use and development of various ROS scavengers, enzyme inhibitors and antioxidant enzymes. Moreover, many *in vitro* studies employ the use of high levels of ROS and thus do not represent the physiological fine-tune modulatory effects of ROS *in vivo*.

In consideration of antioxidant mechanisms, much data indicate that nuclear factor erythroid 2-related factor-2 (Nrf2), a ubiquitously expressed redox-sensitive transcription factor, is important in initiating the transcription of antioxidant and cytoprotective genes [63]. In the cytoplasm, Nrf2 is bound to its inhibitor Kelch-like ECH-associated protein1 (Keap1), and in response to pro-oxidant stress, oxidative modification of Keap1 dissociates Keap1 from Nrf2, facilitating the nuclear translocation of Nrf2 [64]. Nrf2 binds to antioxidant response elements (AREs) to orchestrate the expression of antioxidant enzymes, including SOD [65], to promote a reduction in ROS. Antioxidants can act by promoting Nrf2 activation [66,67], and deficiencies in Nrf2 factor have been associated with increased inflammation and carcinogenesis [68]. The enzyme heme oxygenase-1 (HO-1) is a key Nrf2 gene target, and a large body of data indicate that HO-1 possesses diverse antioxidant and anti-inflammatory capacity [69]. HO-1 is an enzyme that catalyses the degradation of heme and is induced under conditions of oxidative stress [70]. Importantly, HO-1 has the proclivity to negate the production of ROS [71] and inflammatory mediators [72], making HO-1 inducers potential therapeutic targets in disease [73].

## ROS and oxidative signalling

While some studies suggest that oxidative stress plays a critical role in the progression of diseases including cancer, diabetes and CNS disorders [1–5,74], many studies have failed to demonstrate that the therapeutic effects of antioxidants translate to a clinical setting [75]. Indeed, although antioxidants are believed to wield anti-inflammatory effects, their assessment as therapeutics in both human and rodent studies has provided contradictory findings [76–79]. On the contrary, the use of pro-oxidant molecules, such as phytol (NOX2 activator and vitamin E precursor), can improve, or even prevent, ongoing inflammation in animal models [80]. Considering this, the controlled production of ROS is recognized to exert crucial effects in regulating biological functions and cell signalling. This occurs via the rapid generation and removal of ROS within defined cell compartments, thus avoiding sustained signalling and/or oxidative stress [28]. Contrary to previous interpretation, ROS signalling may regulate the inflammatory response.

As previously described, to eliminate invading pathogens, phagocytic cells produce ROS into phagolysosomes following NOX2 activation, and this results in a pH increase within the vesicles, triggering the activation of proteolytic enzymes which in turn destroys the engulfed pathogens/debris [8,81]. Radicals that escape the vesicle membrane into the cytoplasm are rapidly eliminated by the cell antioxidant mechanisms. It is important to note that there are some indications that ROS production can regulate the activation of the NLRP3 inflammasome during inflammation [82,83]. The inflammasome consists in a protein complex that can activate caspase-1 to control the production of IL-1β, a classic pro-inflammatory cytokine [84]. Although ROS may regulate NLRP3 inflammasome activation, it appears to be independent of NOX2 activation. Other known NLRP3 inflammasome activators include innate stimuli (e.g. pathogens and environmental insults) and particulate adjuvants (e.g. alum, silica and urate crystals) [85].

As previously discussed, NO plays a role on oxidative signalling mechanisms [43], in addition to its important role in modulating physiological functions such as neurotransmission, vasodilation and immunomodulation [86]. NO is synthesized on demand and is produced by one of the three known isoforms of nitric oxide synthase (NOS): neuronal NOS (nNOS), inducible NOS (iNOS) and endothelial NOS (eNOS). The nNOS and eNOS isoforms are constitutively present in neuronal and endothelial cells, respectively [86], while iNOS expression is inducible and regulated at the transcriptional level, particularly in inflamed tissues [87]. As mentioned previously, the reaction of O_2_^•−^ with NO can generate ONOO^−^ [43] (Figure 1), which can contribute to generalized oxidative/nitrosative damage, including DNA fragmentation and lipid degradation.

Oxidative stress ensues when ROS are produced in high quantities without control, and ROS production plays a key role in neuroimmune disorders, resulting in several harmful nonspecific events such as DNA laddering and lipid peroxidation. While some radicals react rapidly and rarely escape the cell membrane (e.g. O_2_^•−^), other more stable radicals can cross the cell membrane and diffuse to adjacent tissues (e.g. H_2_O_2_) [88]. Moreover, O_2_^•−^ gives rise to HO^•^ and perhydroxyl (HOO^•^) radicals [29,89], with data indicating that the HO^•^ radical can inactivate the mitochondrial enzyme pyruvate dehydrogenase [90], depolymerize gastrointestinal mucin [91] and inflict oxidative DNA damage [92]. The HOO^•^ is also highly deleterious, initiating lipid peroxidation [93], disturbing membrane permeability [94] and demonstrating toxicity [95]. Such events may be associated with mutagenesis in chronic intestinal inflammation [96] and in addition to intestinal inflammation, much data link the production of ROS to the pathology of a range of inflammatory diseases such as RA, atherosclerosis, MS and IBD [1–5]. The key role of ROS in physiological processes adds to the complexity of targeting ROS production therapeutically, and the balance between ROS elimination and generation may be key in terms of the management of disease.

## Cannabinoids

The introduction of medicinal cannabis, and cannabis-based therapeutics, to mainstream medicine has proved a controversial topic, although medicinal benefits of the *Cannabis sativa* L. (*C. sativa*) plant have been recorded for centuries. Indeed, evidence exists for medicinal use of cannabis dating back to the fourth century when used in the treatment of a broad range of ailments [97]. Much public reticence with the introduction of cannabinoids to a medical setting are associated with the recreational use of cannabis, the euphoric effects of the drug and the evidence linking cannabis abuse with psychosis [98]. With the discovery and characterization of many compounds in *C. sativa*, in addition to an increase in our understanding of cannabinoid pharmacology and toxicology, selective cannabinoid-based therapeutics continue to make advancement in pre-clinical and clinical studies.

To date, a large body of data suggest that cannabinoids have therapeutic properties, alleviating symptoms of several CNS disorders. Indeed, cannabinoids can mitigate inflammation, reduce CNS spasticity, alleviate neuropathic pain, and a body of evidence indicates that cannabinoids provide neuroprotection following injury or inflammation in the CNS [99,100]. The most well-known cannabinoids are the phytocannabinoids synthesized by *C. sativa*, including THC, a euphoric component of the plant, in addition to CBD, cannabinol (CBN), cannabichromene (CBC) and cannabigerol (CBG), which are considered non-euphoric cannabinoids [101]. Cannabinoids also include a class of lipid messengers known as endogenous cannabinoids (eCBs), with *N*-arachidonoylethanolamine (anandamide; AEA) and 2-arachidonoyl-glycerol (2-AG) representing the most actively studied eCBs [102]. Both AEA and 2-AG are synthesized on demand in the body to mediate diverse physiological functions via interaction with a range of receptors, particularly the G-protein-coupled receptors (GPCRs), CB_1_ and CB_2_ [103]. The system of eCBs and cannabinoid receptors constitutes the eCB system (ECS). In addition, many synthetic agonists, antagonists and inverse agonists for the cannabinoid receptors have been developed and such classes of molecules are under investigation.

## Phytocannabinoids

*C. sativa* is an annual dioecious plant containing a diverse repertoire of botanical cannabinoids commonly known as phytocannabinoids. To date approximately 150 phytocannabinoids have been characterized in the plant [104]. The primary phytocannabinoids are THC and CBD, which represent the most commonly studied phytocannabinoids in experimental and clinical settings, demonstrating a wide range of effects on physiological processes. Other phytocannabinoids currently under investigation include the less well-characterized phytocannabinoids, CBG and CBC. The biosynthetic pathways involved in the generation of the main classes of phytocannabinoids involve the formation of acidic cannabinoids including cannabigerolic acid (CBGA), the precursor of tetrahydrocannabinolic acid (THCA), cannabidiolic acid (CBDA) and cannabichromenic acid (CBCA), which have poor oxidative stability. Following oxidation or decarboxylation, neutral forms of cannabinoids are generated by heating or naturally as the plant ages [105]. Other compounds isolated from *C. sativa* include a variety of terpenes [106]. Indeed, over 200 terpenes have been identified, including the monoterpenes (limonene, α-pinene and linalool) and sesquiterpenes (β-caryophyllene), which share the same biological precursor with phytocannabinoids. Terpenes produced by the plant are responsible for the plant aroma [107], acting as botanical insecticides and attracting predatory mites [108].

Phytocannabinoids and terpenes accumulate in the secretory cavity of the glandular trichomes in *C. sativa* [109,110] and are present in the highest quantity on the female flower of the plant. Male plants produce lower levels of phytocannabinoids [111]. Glandular trichomes consist of a sac-like cavity packed with secretory vesicles known as glandular hairs. Glandular trichomes of *C. sativa* alter morphology and metabolite content during flower maturation, and phytocannabinoids/terpenes are found on the calyx and the underside of anthers of flowers, leaves and bracts [109]. Trichomes rupture due to environmental stress or damage (due to high temperatures and herbivorous consumption), resulting in the release of phytocannabinoids and terpenes as a noxious, sticky liquid on the plant surface.

## The ECS

The activity of phytocannabinoids was initially considered to result from their proclivity to fluidise membranes. However, in the early 1990s the cannabinoid receptors CB_1_ [14] and CB_2_ [15], the receptors of the ECS, were identified. Both receptors are GPCRs and are responsible for many of the effects of cannabinoids on physiological systems. CB_2_ is abundantly expressed in immune tissues [112] and is responsible for many immunomodulatory effects, while CB_1_ expression is predominantly confined to the CNS, with expression also identified in peripheral tissues including the heart, reproductive organs and thymus [112]. Indeed, CB_1_ is considered one of the most highly expressed GPCRs in the brain [113]. Under normal physiological conditions CB_2_ exhibits low basal expression in the CNS, with evidence indicating that this receptor is expressed in the brain stem [114] and on hippocampal pyramidal neurons [115]. Importantly, in pathological conditions, data suggest that the expression of the CB_2_ receptor is up-regulated, particularly on microglial cells [116]. Cannabinoids vary in their affinity for the cannabinoid receptors. Indeed, THC is a partial agonist for both CB_1_ and CB_2_ [117], whereas CBD has low affinity for both CB_1_ and CB_2_ [117,118].

The discovery of receptors that mediate the cellular action of components of *C. sativa* was followed by the subsequent identification of the eCBs, AEA [119] and 2-AG [120,121], again in the early 1990s. eCBs vary in their affinities for the cannabinoid receptors; AEA has relatively high affinity for CB_1_ but little to no affinity for CB_2_, while 2-AG is an agonist at both CB_1_ and CB_2_ [122]. AEA and 2-AG are non-charged, hydrophobic lipid molecules that can act to control neurotransmission [123] and are produced on demand in response to an increase in intracellular Ca^2+^ [124–126]. Basal concentrations of 2-AG in brain tissue are 170 times greater than AEA [127]. Furthermore, AEA is a member of the family of *N*-acylethanolamines, other members of which include the eCB-like compounds, palmitoylethanolamide (PEA) and oleolylethanolamide (OEA), both of which possess diverse physiological functions [128–130].

Although CB_1_ and CB_2_ are considered classic cannabinoid receptors, much evidence suggests the existence of further receptor targets for cannabinoids, including nuclear PPARs, GPR55 and the TRPV1. Although GPR55 shares a low amino acid sequence homology with both CB_1_ and CB_2_, data indicate that GPR55 is a receptor for cannabinoid ligands including AEA, PEA, THC and CBD [16,17,131–135]. TRPV1 is a cationic channel receptor dependent on intrinsic and extrinsic calcium concentrations, and evidence indicates that TRPV1 is a target for CBD, 2-AG and AEA [18,136,137]. This receptor exists largely on sensory neurons and cannabinoid activity through this receptor has shown significant analgesic potential [138]. Several studies have also shown that CBD targets PPAR-γ [13,19,139,140], and it should also be noted that PEA and OEA can exert their effects via PPARs [141–143]. In terms of CBD, further CB_1/2_-independent cellular targets include serotonin (5-HT) receptors [19,20,144,145] and both μ- and δ-opioid receptors [146].

## Synthetic cannabinoids

Multiple synthetic cannabinoid compounds have been developed with the aim to pharmacologically target specific aspects of the ECS and to facilitate the development of cannabinoid therapeutics [147]. Firstly, the compound WIN55,212-2, an aminoalkylindole derivative, with affinity for both cannabinoid receptors, but binding to CB_2_ with higher affinity, represents one of the most actively studied synthetic cannabinoids [148,149]. Indeed, research on this compound has shown that WIN55,212-2 is an analgesic in rodent models of neuropathic pain [150] and mechanical allodynia [151], while topical administration of WIN55,212-2 was shown to reduce intraocular pressure in patients with glaucoma [152]. HU-210 is another synthetic cannabinoid agonist that has been extensively researched. HU-210 is a high affinity CB_1_/CB_2_ receptor agonist [153,154] with agonist activity also at GPR55 [16]. This cannabinoid has been shown to be protective in models of experimental colitis [155] and photoreceptor degeneration [156]. The canabinoid agonist CP55,940 shares biochemical properties with THC, demonstrates high affinity for CB_1_ and CB_2_ [157,158] and is also a GPR55 agonist [16]. CP55,940 has been shown to mitigate tumour-evoked hyperalgesia in murine models in a dose-dependent manner [159] and also possesses antioxidant capacity in neutrophils [160]. The JWH family of synthetic cannabinoids have also been shown to be effective agonists of the cannabinoid receptors. Indeed, evidence indicates that JWH-133, a selective CB_2_ agonist [161], ameliorates spasticity in murine MS [162] and prevents microglial cell activation and inflammation following exposure to β-amyloid [163]. Finally, ACEA is a well described synthetic CB_1_ agonist and analogue of AEA [164]. ACEA has the proclivity to protect ischemic neurons from oxygen-glucose deprivation/reoxygenation and middle cerebral artery occlusion [165].

Overall, there is a large array of synthetic cannabinoid ligands currently employed in cannabinoid pharmacological research. Such synthetic cannabinoid ligands are useful pharmacological tools that may be used to identify therapeutic avenues in the ECS.

## Antioxidant and anti-inflammatory effects of cannabinoids: focus on CBD

**Figure 2:**
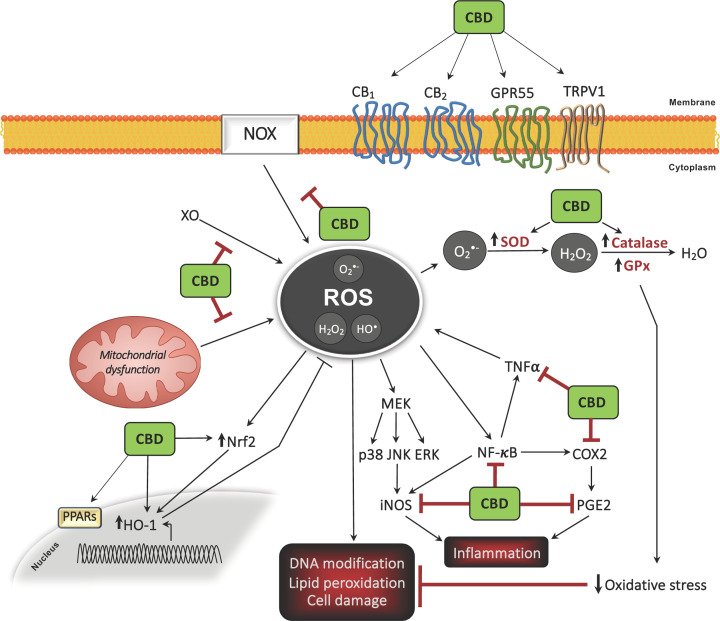
Overview of the antioxidant propensity of CBD

The antioxidative and anti-inflammatory properties of cannabinoids across a variety of tissue types and cellular models have been studied for several decades and are well described [166]. A summary of the antioxidant capacity of CBD is presented in Figure 2. In particular, much evidence has highlighted that CBD, the major non-euphoric phytocannabinoid in *C. sativa*, has an array of anti-inflammatory effects, with proclivity to modulate oxidative processes in neuropathic and inflammatory models [167]. As discussed previously, CBD can act through cannabinoid receptor-dependent and -independent mechanisms, and demonstrates minimal agonist activity (and very low affinity) for both CB_1_ and CB_2_ [117,118]. Furthermore, CB_1/2_-independent mechanisms of action for CBD have also been identified, including PPAR-γ [13,19,139,140], TRPV1 receptor [18], GPR55 [16], 5-HT receptors [19,20,144,145] and μ-/δ-opioid receptors [146]. Much data suggest that CBD has antioxidant capacity, and CBD has been shown to reduce oxidative metabolism in polymorphonuclear leukocytes [168] and H_2_O_2_-treated nucleus pulposus cells [169], and furthemore reduces oxidative stress parameters in aged pancreatic cells [170]. In addition, CBD has the proclivity to improve cell viability following H_2_O_2_ treatment [169]. In addition, data from Barichello and colleagues (2012) indicate that CBD reduces host immune responses and exerts an anti-inflammatory effect (reduced cortical TNF-α) in the CNS of rats exposed to intrathecal *Streptococcus pneumoniae* [171]. This is significant given the key role played by TNF-α in the CNS, particularly in events associated with neuroinflammation, neurodegeneration and neurogenesis [172–174]. Broadly, CBD has an inhibitory effect on TNF-α expression in an array of inflammatory models, which has been recently reviewed by Nicols and Kaplan (2020) [175]. Interestingly, CBD attenuates neural production of ROS following cadmium chloride treatment, in a manner similar to vitamin E (α-tocopheryl acetate) [176], and evidence also suggests that it is more neuroprotectve than ascorbate and α-tocopherol against glutamate toxicity [177]. Owing to its functional and pharmacological diversity, and evidence of is comparable antioxidant capacity to known antioxidants, CBD is an attractive agent for therapeutic immunomodulation and has been studied in conditions including intractable epilepsy [26], Huntington’s disease [178] and schizophrenia [179], amongst others.

## Antioxidant mechanisms of action of CBD

### Regulation of antioxidants

A body of research evidence indicates that CBD modifies redox balance by altering the level and activity of antioxidant molecules (Figure 2). Indeed, CBD has been shown to target the regulation of redox-sensitive transcription factors such as Nrf2 in microglia [180], keratinocytes [181] and endothelia [182], which is important given the key role of Nrf2 in initiating the transcription of antioxidant and cytoprotectve genes [63]. Indeed, recent data have shown that CBD can target the expression of Keap1 and Nrf2 in a pulmonary arterty smooth muscles cells, which may contibute to its antioxidant effect in a model of pulmonary arterial hypertension [183]. CBD has also been shown to regulate the expression of the inducible antioxidant enzyme HO-1 in keratinocytes [184], adipose tissue-derived mesenchymal stem cells [185], neuroblastoma cells [186] and smooth muscle [187], which may impact the proclivity of this phytocannabinoid to regulate cellular ROS levels. Indeed, CBD significantly increases HO-1 mRNA and protein expression in human umbilical artery smooth muscle cells in a time- and concentration-dependent manner independent of CB receptors, and this effect was reversed via glutathione precursor N-acetyl cysteine (NAC), indicating the participation of ROS signalling in this process [187]. Similar effects of CBD were determined in human umbilical vein endothelial cells [182]. Data elsewhere indicate that CBD can also regulate the activity of SOD and the enzymatic activity of Cu-, Zn- and Mn-SOD, which metabolize superoxide radicals [181,188]. The vasorelaxant effects of CBD are reduced by a SOD inhibitor, suggesting that CBD acts via SOD to promote its vascular actions [189]. CBD also attenuates hippocampal oxidative damage post-oxygen–glucose-deprivation/reperfusion injury, up-regulating GSH levels and simultaneously increasing SOD1 and GPx activity following injury [190]. In support of this, *in vivo* administration of CBD attenuates the reduction in reduced/oxidized glutathione ratio (GSH/GSSG) in myocardial tissue of diabetic mice [188], and furthermore prevents GSH depletion in cardiac tissue following doxorubicin cardiotoxicity [191].

### Free radical scavenging capacity

A body of data indicates that CBD demonstrates intrinsic free radical scavenging capacity (Figure 2). Indeed, CBD has been shown to ameliorate LPS-induced ROS production in microglia [192]. Furthermore, CBD inhibits mitochondrial superoxide generation in high glucose-stimulated human coronary endothelial cells [193] and reduces mitochondrial ROS generation following hippocampal oxidative damage post-oxygen–glucose-deprivation/reperfusion injury [190]. Neuroprotective effects of CBD have been shown in models of retinal neurotoxicity whereby CBD directly mitigates *N*-methyl-D-aspartate (NMDA)-mediated oxidative stress by potentially targeting the production of nitrotyrosine, a product of tyrosine nitration [194]. Similarly, oligodendrocyte progenitor cells and keratinocytes are protected from H_2_O_2_-induced cell death by CBD, with this phytocannabinoid demonstrating ROS scavenging properties against H_2_O_2_-induced ROS in the progenitor cells and keratinocytes [195,196]. CBD was recently shown to exert a similar effect on H_2_O_2_-induced ROS in intestinal cell monolayers [197]. Furthermore, data from Branca and colleagues (2019) indicate that CBD attenuates neural ROS production following cadmium chloride exposure in a manner similar to α-tocopheryl acetate [176], and CBD also dose-dependently reduces β-amyloid-induced ROS production in neurons [198]. In parallel, CBD has been shown to ameliorate cisplatin-induced production of renal nitrotyrosine in a model of nephrotoxicity [199] and has also been shown to dose-dependently reduce tert-butyl hydroperoxide-induced ROS production in keratinocytes [184]. In support of this, CBD reduces chemotactic peptide-induced ROS production in polymorphonuclear leukocytes [168] and furthermore *in vivo* administration of CBD ameliorates the production of ROS, in addition to lipid peroxides, in myocardial tissue of diabetic mice [188]. Finally, recent data from Baeeri and colleagues (2020) indicate that CBD attenuates age-related increases in ROS production in pancreatic islets [170], overall indicating the proclivity of CBD to exert free radical scavenging capacity in response to a multitude of stimuli.

### Redox balance

CBD principally affects redox balance via intrinsic mechanisms. Indeed, evidence indiates that CBD interrupts free radical chain reactions, transforming free radicals into more inert molecules via the electrophilic aromatic molecular region and hydroxyl groups of its phenol ring [200]. Using cyclic voltammetry, Hampson et al. (1998) demonstrated that CBD donated electrons at a similar potential to known antioxidants, and using the iron-catalysed ROS production system (Fenton reaction), CBD was shown to prevent hydroperoxide-induced oxidative damage in neurons [177]. Again, using cyclic voltammetry, Hamelink and colleagues (2005) demonstrated that CBD is a comparable antioxidant to the common antioxidants tocopherol and butylated hydroxytoluene [201], with data indicating that CBD can reduce ROS production by chelating transition metal ions involved in the Fenton reaction [202]. Neuroprotective effects of CBD have also been shown following anti-Yo-associated paraneoplastic cerebellar degeneration, where data indicate that CBD minimizes the downgrading of the mitochondrial membrane potential induced by anti-Yo in a manner similar to the ROS scavenger butylated hydroxytoluene, although simultaneously potentiated Yo-induced ROS production [203]. CBD has also been shown to protect hippocampal neurons from energy stress via modulation of glucose consumption by activation of the pentose-phosphate pathway in an oxygen–glucose-deprivation/reperfusion injury model [190].

### Indirect antioxidant actions on XO and NOX

In addition to its intrinsic antioxidant function, CBD alters oxidative metabolism through more indirect mechanisms to modulate downstream mediators of oxidative stress (Figure 2). Indeed, Atalay and colleagues (2020) recently demonstrated that CBD attenuates XO activity in keratinocytes exposed to UVB irradiation and H_2_O_2_ [204]. CBD has been shown to reduce the expression of the superoxide generators RENOX (NOX4) and NOX1 in a mouse model of cisplatin-induced nephrotoxicity [199], and in a comprehensive study, Rajesh et al*.* (2010) identified the proclivity of CBD to attenuate diabetes-induced mRNA expression of NADPH oxidase subunits (p22phox, p67phox and gp91phox) in myocardial tissue using a model of diabetic cardiomyopathy [188]. Elsewhere, CBD has been shown to attenuate LPS-induced intracellular NADPH synthesis in microglia [192], and similarly, *in vivo* administration of CBD reduces the hepatic expression of the NADPH NOX2 isoforms p67phox/gp91phox and nitrosative stress in ethanol-fed mice, while reducing oxidative burst in neutrophils isolated from the livers of ethanol-fed mice [205]. CBD has also been shown to reduce nitrosative stress in cardiac tissue following doxorubicin-induced cardiac injury in rats, promoting a reduction in nuclear factor-κB (NF-κB), Fas ligand, caspase-3 and TNF-α in cardiac tissue following doxorubucin administration [191]. Similarly, CBD attenuates H_2_O_2_-induced COX2 and iNOS in nucleus pulposus cells [169], attenuates iNOS expression in the myocarium of diabetic mice [188] and in rats following doxorubicin cardiotoxicity [191], and furthemore attenuates NO expression in paw tissue following sciatic nerve injury and intraplantar injection of complete Freund’s adjuvant [167]. In parallel, Rajesh and colleagues (2007) demonstrated that CBD blunts iNOS expression in high glucose-stimulated human coronary endothelial cells [193], and data elsewhere indicate that CBD inhibits cisplatin-induced renal iNOS in a mouse model of nephrotoxicity [199]. CBD has also been shown to ameliorate plasma levels of prostaglandin E_2_ (PGE_2_) in animal models of neuropathic and inflammatory pain, using sciatic nerve constriction and intraplantar injection of complete Freund’s adjuvant, respectively [167]. By lowering ROS levels, CBD therefore protects non-enzymatic antioxidants, preventing their oxidation. Recent evidence also suggests that CBD can ameliorate H_2_O_2_-induced IL-1β expression and the expression of other NLRP3 inflammasome-related genes, and this area warrants full investigation [196].

### Pro-oxidant effects of CBD

Despite data indicating that CBD promotes antioxidative metabolism in various cells/systems, conflicting data exist regarding the influence of CBD on redox status. The effects of CBD on cell viability [170,181,195,206–210] and proliferation [187,211] are dose-dependent, and this may also be the case with respect to its antioxidant function. Indeed, in terms of ROS production, CBD has been shown to disrupt mitochondrial integrity and also induce ROS production and apoptosis in human CD14^+^ monocytes in a time-dependent manner [206]. Studies also indicate that CBD elevates intracellular ROS production in several cell systems, including human THP-1 monocytes [207], mouse macrophages [212], breast cancer cells [213] and human glioma cells [214]. Similarly, CBD promotes ROS production to promote apoptosis in leukaemia cells [208], thymocytes [211] and splenocytes [209], and data from Panja and colleagues (2019) indicate that CBD potentiates Yo-induced ROS production in a model of postparaneoplastic cerebellar degeneration treatment [203]. CBD has also been shown to overcome oxaliplatin resistance in human colorectal cancer cells by inducing mitochondrial dysfunction by increasing intracellular ROS and decreasing SOD2 [215]. Data from Gonzalez-Garcia *et al*. (2017) indicate that CBD induces apoptosis of encephalitogenic cells through oxidative stress induction (increase in ROS) in murine MS [210]. CBD has also been shown to increase the oxygen consumption rate and enhance mitochondrial bioenergetics [190], and at high concentrations can promote COX-2 expression in LPS-treated macrophages [216]. In further support of the pro-oxidant capacity of CBD, it is also important to note that CBD has been shown to increase the expression of the NOX4 and p22phox NAP(P)H oxidases [208], and also enhances the production of the p47 subunit of pro-oxidative NADPH oxidase in endotoxin-treated neutrophils from healthy subjects [217]. Finally, CBD promotes a reduction in GSH in splenocytes [209] and has been shown to deplete GSH in human gliomas [214].

Overall, CBD modulates redox function at multiple levels and a variety of downstream effects are presented in the literature. These pro- and antioxidant functions may be cell- and model-dependent, and may also to be influenced by the dose of CBD delivered, the duration of CBD treatment and the underlying pathology.

### Cannabis-based therapeutics

Cannabinoid-based therapeutics are currently approved for a range of symptoms in various disorders; anorexia associated with loss of appetite in acquired immunodeficiency syndrome (AIDS), chemotherapy-associated nausea, spasticity in people with (pw)MS and the treatment of seizures in Lennox-Gastaut and Dravet syndromes. The therapeutics include Epidiolex®, Sativex®, Marinol® and Cesamet®, and such cannabinoid-based therapeutics are used as treatment strategies in those patients where conventional treatments are ineffective.

Of particular relevance to this review, the CBD-based therapeutic in the form of Epidiolex® has recently entered the clinic in the management of epileptic seizures. Epidiolex®, a purified solution of CBD oil formulated for oral administration [24], is a medication approved by the US Food and Drug administration (FDA) and the European Medicines Agency (EMA) for patients with Lennox–Gastaut Syndrome and Dravet syndromes. CBD demonstrates efficacy in reducing convulsive seizure frequency in double-blind placebo-controlled trials [218], and in a prospective open-label study assessing the efficacy of Epidiolex® in patients with treatment resistant epilepsy, Epidiolex® improved the severity and frequency of seizures, and this was sustained for up to 48 weeks of treatment [219]. Devinsky and colleagues (2018) also demonstrated that 10 and 20 mg/kg doses of Epidiolex® (administered as two doses per day over a 14-week period) ameliorated drop seizures in patients with Lennox–Gastaut syndrome, when compared with placebo control cases [25].

In a second cannabis-based therapeutic, CBD is combined with THC as an oromucosal spray (Sativex®), developed to manage spasticity in pwMS [220]. Sativex® can be used as an add-on therapy to ameliorate MS symptoms, with each 100 μl Sativex® actuation delivering 2.5 mg CBD and 2.7 mg THC, in addition to other constituents [221,222]. In terms of effectiveness in ameliorating spasticity, data indicate that the majority of pwMS that administer Sativex® experience an improvement in spasticity score within a 4-week period [223].

Finally, both Marinol® (a pharmaceutical formulation of synthetic THC) and Cesamet® (a synthetic analogue of THC) are FDA approved cannabinoid therapeutics that can be administered orally in tablet form for the managment of chemotherapy-induced nausea/emesis in patients who are refractory to other antiemetics [224,225]. Marinol® is also indicated for anorexia associated with loss of appetite in AIDS patients [225], and data suggest that Cesamet® has efficacy as an analgesic in chronic non-cancer pain [226].

It should be noted that given that adverse effects may be associated with cannabinoids, certain cannabis-based therapeutics are contraindicated in patients with a history of psychotic illness, schizophrenia, substance abuse and also in pregnancy and patients who are breast feeding. Certain cannabis-based therapeutics are also not recommended for use in certain age groups.

## Conclusions

It is clear that cannabinoid use in the clinical setting has a wide variety of uses in terms of ameliorating symptoms of neurological disease, such as seizure disorders and MS, and further cannabis-based therapeutics are in the pipeline for a range of disorders. Epidiolex®, a purified solution of CBD, is in the clinic for the management of epileptic seizures. Given the recent advancements in the clinical development of CBD, this review has focussed on the signalling targets for this phytocannabinoid, and in doing so, aimed to outline both the complex role played by ROS in disease processes, in addition to the key role of ROS in regulating biological functions. It is clear that ROS are integral players in neuroinflammation, peripheral immune responses and physiological processes, and herein we have summarized evidence that CBD readily targets oxidative signalling and ROS production. Further research is required to fully understand the interplay of this phytocannabinoid with oxidative signalling. The balance between the ability of CBD to regulate both the generation and elimination of ROS may govern its effectiveness in impacting the symptoms of disease.

## Abbreviations

2-AG: 2-arachidonoyl-glycerol
5-HT1A: 5-hydroxytryptamine receptor subtype 1A
AEA: anandamide
AIDS: acquired immunodeficiency syndrome
ARE: antioxidant response element
CAT: catalase
CBC: cannabichromene
CBCA: cannabichromenic acid
CBD: cannabidiol
CBDA: cannabidiolic acid
CBG: cannabigerol
CBGA: cannabigerolic acid
CBN: cannabinol
CGD: chronic granulomatous disease
COX-2: cyclooxygenase-2
eCB: endocannabinoid
ECS: eCB system
EMA: European Medicines Agency
eNOS: endothelial NOS
FDA: US Food and Drug administration
GPCR: G-protein-coupled receptor
GPR55: G-protein-coupled receptor 55
GPx: glutathione peroxidase
GSH: glutathione
GSSG: glutathione disulfide
HO-1: heme oxygenase-1
IBD: inflammatory bowel disease
IFN: interferon
IL: interleukin
iNOS: inducible NOS
Keap1: kelch-like ECH-associated protein1
LO: lipoxygenase
LPS: lipopolysaccharide
MS: multiple sclerosis
NAC: N-acetyl cysteine
NF-κB: nuclear factor-κB
NMDA: *N*-methyl-D-aspartate
nNOS: neuronal NOS
NO: nitric oxide
NOS: nitric oxide synthase
NOX2: NADPH oxidase 2
Nrf2: nuclear factor erythroid 2-related factor-2
OEA: oleolylethanolamide
PEA: palmitoylethanolamide
PGE_2_: prostaglandin E_2_
PPAR: peroxisome proliferator-activated receptor
pwMS: people with MS
RA: rheumatoid arthritis
ROS: reactive oxygen species
SOD: superoxide dismutase
THC: tetrahydrocannabinol
THCA: tetrahydrocannabinolic acid
TLR: toll-like receptor
TNF-α: tumour necrosis factor-α
TRPV1: transient receptor potential cation channel subfamily V member 1
XO: xanthine oxidase
